# Improvement of Workplace Environment That Affects Motivation of Japanese Dental Hygienists

**DOI:** 10.3390/ijerph18031309

**Published:** 2021-02-01

**Authors:** Yuko Yamamoto, Yoshiaki Nomura, Ayako Okada, Erika Kakuta, Naomi Yoshida, Noriyasu Hosoya, Nobuhiro Hanada, Noriko Takei

**Affiliations:** 1 Department of Endodontology, Tsurumi University School of Dental Medicine, Kanagawa 230-8501, Japan; yamamoto-y@tsurumi-u.ac.jp (Y.Y.); hosoya-n@tsurumi-u.ac.jp (N.H.); 2 Department of Translational Research, Tsurumi University School of Dental Medicine, Kanagawa 230-8501, Japan; hanada-n@tsurumi-u.ac.jp; 3 Department of Operative Dentistry, Tsurumi University School of Dental Medicine, Kanagawa 230-8501, Japan; okada-a@tsurumi-u.ac.jp; 4 Department of Oral Microbiology, Tsurumi University School of Dental Medicine, Kanagawa 230-8501, Japan; kakuta-erika@tsurumi-u.ac.jp; 5 Japan Dental Hygienists’ Association, Tokyo 169-0072, Japan; yoshida.ohce@tmd.ac.jp (N.Y.); nori@pm-ms.tepm.jp (N.T.)

**Keywords:** dental hygienist, workplace environment, item response theory (IRT), decision analysis, factor analysis

## Abstract

Dental hygienists are in high demand due to insufficient workforce and a lack of an effective reinstatement support system. We investigated the reasons for willingness to work by analyzing the survey results of the employment status of Japanese dental hygienists conducted by Japan Dental Hygienists’ Association. In total, we mailed 16,113 questionnaires to all members of the association (response rate 53.4%). We carried out statistical analysis to determine the specific items to improve the hygienists’ working environment. Fourteen factors of working conditions that they wish to improve were determined. Structural equation modeling showed that a path, “Reduction of work volume”, “Reduction of working hours” and “Increased number of holidays” were higher than other items. A decision analysis demonstrated that most of the respondents answered “Yes” to “Improvement in working conditions including higher salary” out of those who answered, “Strongly disagree” for “Do you feel that dental hygienist work is rewarding?”. Improving workplace environment is integral to keeping high levels of work motivation and a low turnover rate. Most of the hygienists wish for a salary raise among all the conditions. The transition from conventional work styles to non-conventional flexible working patterns is needed.

## 1. Introduction

Japan has an aging rate of 28.4% in 2019 and is categorized as a super-aged society. The share of elderly generation of the total population is expected to continue growing [1]. Dental hygienists play an important role in the extended dental healthcare service [2]. As growing number of seniors are aware of the importance of oral well-being and avoiding getting frail, far-reaching dental healthcare service is required in the Japanese super-aged society. Dental hygienists are expected to contribute considerably to maintaining oral function for the elderly, including swallowing function training and stimulation of saliva secretion [3,4]. Dental hygienists working at hospitals and residential facilities make a significant contribution in tackling the suffering, morbidity, and mortality associated with respiratory infections [5,6]. Thus, the demand for dental hygienists has been rapidly increasing in Japan, however, this demand is not met due to insufficient workforce of dental hygienists and a lack of effective reinstatement support systems. The number of licensed dental hygienists was estimated to be approximately 250,000, while the number of working dental hygienists was only 116,299 as of 2014 [7]. According to a survey conducted by Japan Dental Hygienists’ Association, the reasons for resignation were not only marriage, childbirth, or childcare, but pursuing other interests in fields other than dentistry [8].

Motivation plays a key role in driving employees towards achieving their goals and organizational goals which lead to serving satisfaction in employees. Motivation is needed for all employees in an organization including dental practices to be productive. Motivation is not always based on financial rewards, however, combinations of non-financial rewards methods can also be used to derive the best out of employees [9]. In the field of dental hygiene in Japan, acquiring and brushing up a variety of technical skills enhances job and career satisfaction which contributes positively to well-being of workers and high employee retention rate [10,11,12].

On the other hand, dental hygienists encounter numerous challenges at work, facing problems such as a heavy workload, job stress, low wages, overtime, a lack of practical skills and job security, and finding a long-term career fulfillment or job satisfaction [13,14,15,16,17,18,19].

The working environment including employee benefits needs thorough reconsideration for the purpose of keeping and attracting more workforce [16,17]. At this moment the exact reasons that actually have an impact on the hygienists’ willingness to work are not yet elucidated. A statistical analysis of environmental factors extracted from the survey may help develop solutions to this issue.

Only a few researches have demonstrated recruitment of hygienists and they are region-specific, descriptive or not fully analytic [20,21]. Moreover, their sample sizes were small and insufficient to represent a whole picture of the current situation of the Japanese dental hygienists.

In this study, we investigated the specific reasons that contribute to the willingness to work among Japanese dental hygienists by analyzing the survey results of the Employment status of Japanese dental hygienists which was conducted by Japan Dental Hygienists’ Association. In addition, we attempted to determine the primary reason that had a major impact on their willingness to work.

## 2. Materials and Methods

### 2.1. Survey Method

Japan Dental Hygienists’ Association has been conducting a survey, the Employment status of Japanese dental hygienists once every 5 years since 1981. A total of 16,113 questionnaires were sent to all members of the association on 30 September 2014 by mail with a return envelope. Respondents filled out the questionnaires on 31 October and sent them back by no later than 30 November.

### 2.2. Questionnaire

The questionnaires used in this study consisted of 94 major items concerning demographic factors, employment situation, contents of work, willingness to work, etc. Among these items, two items concerning motivation of Japanese dental hygienists: “Do you feel that dental hygienist work is rewarding?”, “Do you wish to continue working as a dental hygienist?” and eight other items related to attractive points of the work were selected for analysis. The eight items include “Do you think that dental hygienist work is to maintain people’s health?”, “Do you think that dental hygienist work can contribute to people and the community?”, “Do you think that being a dental hygienist is good to yourself?”, “Do you think that dental hygienist work requires a high level of expertise?”, “Do you think that dental hygienist work provides employment security?”, “Do you think that income is assured?”, “Do you think that only nationally licensed dental hygienists are hired to work as dental hygienists?”, and “Do you think that women are better suited to working as dental hygienists?”.

### 2.3. Statistical Analysis

Descriptive statistics of the frequency of the items and cross tabulations with age groups were summarized. Chi-square tests were used for the items described above.

Item response theory (IRT) was applied to calculate the item discriminations, and item difficulties to figure out attractive points of the work. Item response curves and item information curves were graphically illustrated. IRT analysis were carried out by R software Ver.3.50 with irtoys and LTR package by following formula:

(1) $$\left(U_{i,j,}|\theta_{i},a_{j},b_{j}\right)=\frac{exp\left(1.76a_{j}\left(\theta_{i}-b_{j}\right)\right)}{1+exp\left(1.76a_{j}\left(\theta_{i}-b_{j}\right)\right)}$$

Factor analysis with varimax rotation was carried out to make the latent variables underlying the response for the items.

The relationship between motivation of the hygienists and attractive points of the work were analyzed by structural equation modeling (SEM). Analysis was carried out by AMOS Ver. 24.0 (IBM, Tokyo, Japan).

Decision analysis was carried out by classification and regression trees (CART). Two items concerning the motivation of the hygienists: “Do you wish to continue working as a dental hygienist?” and “Do you feel that dental hygienist work is rewarding?” were used for the objective variable and eight items for attractive points of the work listed in Section 2.2 were used for the explanatory variable. SPSS Statistics Ver. 24.0 (IBM, Tokyo, Japan) was used for analysis.

### 2.4. Ethics

This study was approved by the Ethics Committee of the Tsurumi University School of Dental Medicine (approval number: 1544) and conducted in accordance with the Declaration of Helsinki.

## 3. Results

### 3.1. Descriptive Statistics of the Survey

A total of 16,113 questionnaires were sent to all members of Japan Dental Hygienists’ Association, and we received 8932 answers: the number of valid responses were 8932, the response rate was 53.4%. When the survey was conducted, the members of Japan Dental Hygienists’ Association were all women. Frequency table of fourteen items that the hygienists wish to improve was shown in Table S1. Cross tabulations of job satisfaction on age or career as a dental hygienist were shown in Table S2. Among the fourteen items that the hygienists wish to improve, those who answered “Yes” for “Improvement in working conditions including higher salary” were 1955 (51.4%).

### 3.2. Item Response Theory (IRT) Analysis for the Items of Japanese Dental Hygienists Wish to Improve

**Figure 1:**
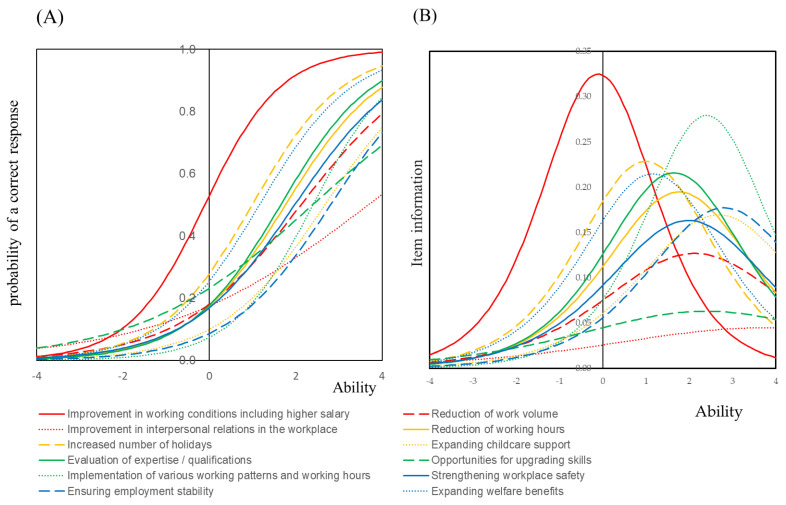
Item response curve and item information curve of Improvement in working conditions.

IRT analysis was carried out for the fourteen items that the hygienists wish to improve. Item response curve and item information curves were graphically illustrated in Figure 1. The model was shown in Table S3.

Among the fourteen items that the hygienists wish to improve, item discrimination of an item, “Improvement in working conditions” was highest and item difficulty was negative which indicates that many Japanese dental hygienists wish to improve this item. As shown in Table S1, the number of hygienists who answered “Yes” for this item was highest (51.4%). As is shown in Figure 1, the item response curve was typical S-shaped, and it was located near the center and the shape of the curve was symmetry and looked like a normal distribution. 

An item of “Implementation of various working patterns and working hours” had high amount of information while the number of subjects who answered “Yes” for this item was not high. Therefore, the item information curve of this item was shifted in the forward direction. Following these items, an item discrimination of “Increased number of holidays”, “Evaluation of expertise/qualifications” and “Expanding welfare benefits” were higher than other items. 

The horizontal axis known as ability according to the item response theory represents the weighted sum of the items that the dental hygienists wish to improve. The vertical axis of the item response curve represents the percentage of those who answered “Yes” to working conditions that they wish to improve.

### 3.3. Motivation of Japanese Dental Hygienists and Improvement of Working Conditions

Furthermore, we analyzed correlations between these items and motivation of the hygienists. Cross tabulations of working conditions that the hygienists wish to improve and their motivation were shown in Table S4.

In terms of the question “Do you feel that dental hygienist work is rewarding?”, all the working conditions that they wish to improve were statistically significant by chi-square tests. In contrast, among the fourteen items that they wish to improve, six factors had statistically significant co-relations.

### 3.4. Structure of Improvement of Working Environment and Motivation

**Figure 2:**
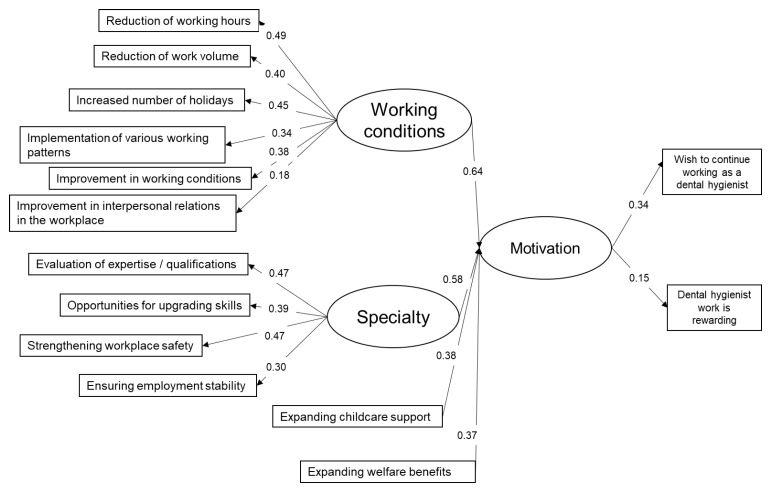
The path diagram of the motivation and working conditions that Japanese dental hygienists wish to improve. A path from “Working conditions” to “Motivation” was higher than that of “Specialty”. Among all observed variables that construct “Working conditions”, coefficients of “Reduction of work volume”, “Reduction of working hours” and “Increased number of holidays” were higher than other items.

Path diagrams play a fundamental role in structural modeling. To construct latent variables, factor analysis was carried out. The result was shown in Table S5. Two latent variables can be constructed. We named these two latent variables “Working condition” and “Specialty”. Two items, “Expanding welfare benefits” and “Expanding childcare support” were independent from other items. To confirm structural validity, structural equation modeling (SEM) was carried out based on the results of factor analysis. The results were shown in Figure 2.

A path from “Working conditions” to “Motivation” was higher than that of “Specialty”. Among all observed variables that construct “Working conditions”, a path concerning quantity of work such as “Reduction of work volume”, “Reduction of working hours” and “Increased number of holidays” were higher than other items. As of latent variables of motivation, a path to “Do you wish to continue working as a dental hygienist?” was higher than that of “Do you think that dental hygienist work is rewarding?”. Among 3807 dental hygienists analyzed in this study, 218 answered “NO” for an item “Do you wish to continue working as a dental hygienist?” and “Strongly disagree” for an item “Do you feel that dental hygienist work is rewarding?”.

### 3.5. The Characteristics of the Dental Hygienists’ Unprofitable Job

**Figure 3:**
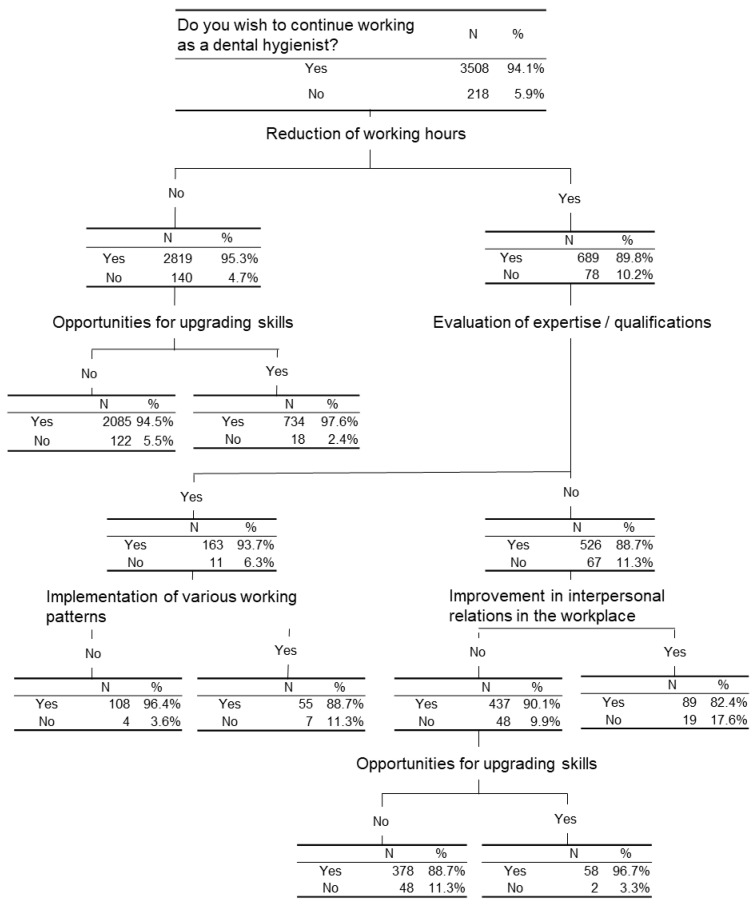
A decision tree to identify subjects who answered “No” for the item of “Do you wish to continue working as a dental hygienist?

**Figure 4:**
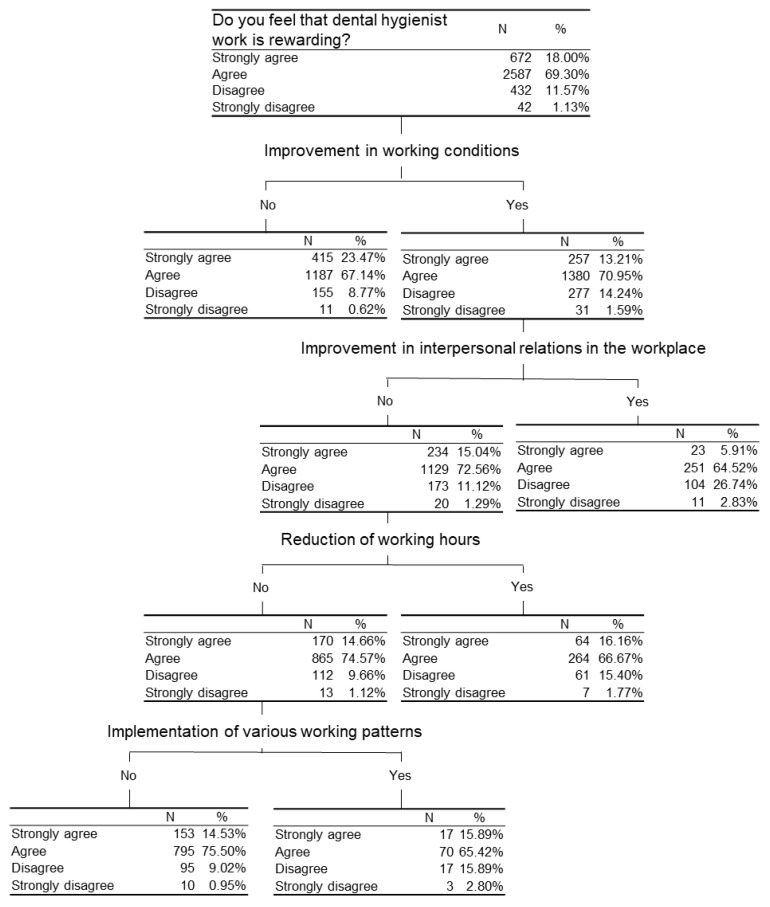
A decision tree to identify subjects who answered, “Strongly disagree” to the “Do you feel that dental hygienist work is rewarding?”.

The characteristics of the hygienists’ unprofitable job was analyzed by decision analysis. Figure 3 demonstrated that 218 dental hygienists answered “No” for the item of “Do you wish to continue working as a dental hygienist?”, and 122 dental hygienists answered “No” for both items of “Reduction of working hours” and “Opportunities for upgrading skills”. Among the 78 hygienists who answered “Yes” for the item of “Reduction of working hours”, 48 answered “No” for “Evaluation of expertise/qualifications”, “Improvement in interpersonal relations in the workplace” and “Opportunities for upgrading skills”. Figure 4 demonstrated that among 42 hygienists who strongly disagree for the item of “Do you feel that dental hygienist work is rewarding?”, 31 answered “Yes” for the item of “Improvement in working conditions”. Among them 11 answered “Yes” for “Improvement in interpersonal relations in the workplace” and 10 answered “No” for the following three items: “Improvement in interpersonal relations in the workplace”, “Reduction of working hours” and “Implementation of various working patterns”.

## 4. Discussion

Though dental hygienists are in high demand in Japan, insufficient labor supply and a lack of effective reinstatement support systems are the problems that have as yet been scarcely dealt with. We investigated the reasons that affect the willingness to work among Japanese dental hygienists and found out that many hygienists wish to improve their working conditions.

In this study, we carried out statistical analysis to determine the specific items to improve the Japanese dental hygienists’ working environment. Fourteen factors of working conditions that they wish to improve were determined, and we found out that the hygienists would most like to improve salary. A number of studies have addressed that dental hygienists are dissatisfied with their salary and they perceived they are underpaid [16,22,23]. In fact, financial rewards play an important role in employee retention and their job satisfaction [16]. We applied Item Response Theory (IRT) for the analysis presented in Figure 1 and Figure 2 and Table S1. IRT was applied to calculate item discriminations, item difficulties, and item guesses for work-related tasks in the previous research [24]. In this study, we made great use of it to assess the survey questionnaires and graphically described the item characteristics. 

We can grasp the whole picture of improvement of working conditions shown in Figure 2. A path from “Working conditions” to “Motivation” was higher than that of “Specialty”. Among the observed variables that construct “Working conditions”, a path concerning quantity of work such as “Reduction of working hours”, “Reduction of work volume”, and “Increased number of holidays” were higher. According to these results, we surmise that Japanese dental hygienists devote their time and energy to work so vigorously that they miss out a chance to acquire holiday. 

In terms of “Specialty”, path coefficients concerning “Evaluation of expertise/qualifications” and “Strengthening workplace safety” were high which indicates that they wish to work in a safe environment to make great use of their high degree of specialization. As of “Expanding childcare support”, no factor was extracted in the factor analysis we performed. This is because these items independently affect “Motivation” of hygienists and childrearing is thought to be a factor that is related to a limited age group. When it comes to “Motivation”, a path coefficient of those who “wish to continue working as dental hygienists” were high in comparison to those who think that “dental hygienist work is rewarding”. Domestically and globally, dental hygienists wish to improve their working environment. In South Korea, Noh et al. [25] concluded that working conditions are factors that are strongly related to job satisfactions and employee turnover rate. Reduction of working hours was an important factor for keeping employees from leaving.

Figure 3 indicates their willingness to continue working as a dental hygienist. 2085 out of 3508 hygienists wish to continue working as a dental hygienist while 122 out of 218 are Not. This group of dental hygienists (2085 + 218) neither expect reduced working hours nor wish to have opportunities for upgrading their practical skills. The results demonstrate that the hygienists in this group accept the status quo and do not expect drastic change in their working style. They seem to believe that there will not be any positive changes in their working hours and opportunities to gain more skills. 

Seventy-eight out of 218 dental hygienists who are Not wishing to continue working as a dental hygienist expect reduced working hours. Forty-eight out of 78 dental hygienists do not seek opportunities to improve practical skills or better interpersonal relations between employees. Evaluation of expertise/qualifications are not requested.

In Figure 3, while 3508 (94.1%) out of all respondents answered “Yes” to “Do you wish to continue working as a dental hygienist?”, most of them did not wish “Reduction of working hours” nor having more “Opportunities for upgrading skills”. This result suggests that the Japanese dental hygienists at any level abide by employment legislations and a sense of the career goals of each personnel is on job security and they rarely think above and beyond. In this sense, hygienists in Japan are likely to be more passive than those in the U.S. In the U.S., dental hygienists can own independent or collaborative practices, provide professional care outside of a traditional dental office and purchase their own equipment and supplies according to the state statutes [26]. A growing number of hygienists feel prepared and competent to perform preventive dental hygiene services without dentist supervision. This move can lead to achieving professional jurisdiction of preventive dental care within the U.S. [27]. We thus assume that those hygienists opt for purchasing their own equipment since they wish to gain exactly what they want. They would end up thinking that they can personalize their working ethics and styles to reduce the pains that may come from their working environment that does not fit them physically and mentally. If the equipment is all theirs, it is relatively easy to establish a healthy workspace environment than working in more than one office as a freelancer. Furthermore, if a hygienist becomes a founder of business, she would never let an office budget determine what she does on a daily basis. Dental hygienists who choose to purchase their own equipment are highly motivated and pride themselves on investing in the future careers and their own physical well-being. The other big advantage of becoming a business owner is that for example, hygienists licensed in California who hold the RDHAP license or special extended care permits (ECP) and those in states such as Oregon and Washington are eligible to deduct business expenses under schedule C [28]. 

Figure 4 demonstrated that of all the subjects who answered, “Strongly disagree” for “Do you feel that dental hygienist work is rewarding?”, most of them answered “Yes” to “Improvement in working conditions including higher salary”. Salary is considered one of the most important factors for motivation of Japanese dental hygienists [16]. A previous study reported that the average per-hour salary of inexperienced dental hygienists was JPY1324.2 ($12.08 U.S.) ± 229.7 ($2.10 U.S.). In 22.9% of dental clinics, the head of dental clinic follows a salary scale based on the dental hygienist’s school background; different salaries are paid for university graduates with a bachelor’s degree and vocational school graduates [29]. In the U.S., however, the average per-hour salary for hygienists is $35.3 U.S. Most of them are now choosing to work part-time [30]. In general, working part-time is likely to entail worse working conditions and lower access to social security and other benefits compared to full-time work. Dental hygienists’ dissatisfaction with low remuneration have been reported in the U.S. [31], Sweden [22], Australia [32,33], and South Africa [34]. When it comes to the Japanese dental hygienists, according to the same survey [8], those hired full-time with or without social benefit shared more than half of all the respondents and the rest were those hired part-time or hired on a day-to-day basis. Part-time hygienists often work for multiple practices to supplement their income. 

In the field of nursing, presence of children may be decreasing the probability of working full-time as a registered nurse [35,36,37]. With dental hygiene being predominantly female profession similar to nursing, balancing work and family is not negligible to be employed on a full-time contract. These employment background should be taken into account when setting a salary and creating pay grades. 

Given the fact that approximately 20–30% of all the dental hygienists participated in the research wish to work reduced hours, reconsideration of working hours could be an effective solution to keep employees from leaving. This study suggested diversification of working styles for dental hygienists. Figure 4 indicates that Japanese dental hygienists who think their work is rewarding do not require any further improvement in working conditions including a salary raise. Those who wish to have improved working conditions do not require better inter-personal relations in workplace between employees or reduction of working hours while they wish to have various working forms and working hours. While many hospitals and dental practices hold conventional working style that firmly fixes workdays and length of working time, diversification of working styles will be needed worldwide especially amid COVID-19. A flexible work shift where job seekers can work on temp or they can work even for a limited time frame such as early morning shift or midnight shift is encouraged to be expanded. 

A locum (locum tenens) is a person who temporarily fulfills vacancies. The term is often used for a physician or clergyman [38]. Just like them, locum tenens dental hygienists can fill in for other hygienists on a temporary basis for a range of a few days to years. When a practice faces temporary staffing shortages due to maternity/paternity leaves, illness, death, vacations, or other causes, they hire locum tenens hygienists to fill those vacancies, make a smooth flow of customers and keep a business running [39,40]. A reliable job matching system needs to be created where employers who seek personals only to fill vacancies without hiring full time workers and job seekers who just need a job that meets their rigid requirements and does not take away all their time and energy are matched in an efficient way.

In summary, improving and creating a healthy work environment is essential for dental hygienists to keeping high levels of work motivation and a low turnover rate. Although perception is different for each person, most of the Japanese dental hygienists wish for a salary raise of all working conditions that they wish to improve. Developing an evaluation system in which hygienists’ performances are objectively and fairly evaluated and workers with high performance can earn a salary raise, encourages their work motivation. Reduction of working hours can reduce employee turnover. The transition from conventional work styles to non-conventional flexible working patterns is needed. 

## 5. Conclusions

Workplace improvement including a salary raise, reduction of working hours and work volume, and increased number of holidays may affect the motivation of the most Japanese dental hygienists. To improve and create a healthy work environment can lead to high levels of work motivation and a low turnover rate. Building an effective performance evaluation system in which hygienists’ performances are objectively and fairly evaluated encourages their work motivation. To implement and manage flexible working patterns is required.

## Data Availability

The data of the present study were used under license for the current study and, therefore, are not publicly available.

## Supplementary Materials

The following are available online at https://www.mdpi.com/1660-4601/18/3/1309/s1, Table S1. Frequency table of fourteen items that Japanese dental hygienists wish to improve; Table S2. Cross tabulations of job satisfaction against age or career as dental hygienists; Table S3. Results of Item response theory analysis by two parameter logistic model; Table S4. Cross tabulations of motivation of Japanese dental hygienists and improvement in working conditions; Table S5. Results of factor analysis for the fourteen items that Japanese dental hygienists wish to improve; Table S6. Cross tabulation of improvement in working conditions against financial reward.
