# Supplementary material for: Improvement of Workplace Environment That Affects Motivation of Japanese Dental Hygienists
Source: Int J Environ Res Public Health. 2021 Feb 1;18(3):1309. doi: 10.3390/ijerph18031309 (PMC7908268; doi:10.3390/ijerph18031309)
Supplement: Supplementary file 1 [file ijerph-18-01309-s001.pdf]

Supplemental materials

Improvement of workplace environment that affects motivation of Japanese dental hygienists

Table S1. Frequency table of fourteen items that Japanese dental hygienists wish to improve

		N	%			n	%
Improvement in working conditions including higher salary	Yes	1955	51.4	Opportunities for upgrading skills	Yes	906	23.8
	No	1779	46.7		No	2828	74.3
	No answer	73	1.9		No answer	73	1.9
Reduction of work volume	Yes	755	19.8	Implementation of various working patterns and working hours	Yes	394	10.3
	No	2979	78.3		No	3340	87.7
	No answer	73	1.9		No answer	73	1.9
Improvement in interpersonal relations in the workplace	Yes	682	17.9	Strengthening workplace safety	Yes	729	19.1
	No	3052	80.2		No	3005	78.9
	No answer	73	1.9		No answer	73	1.9
Reduction of working hours	Yes	768	20.2	Ensuring employment stability	Yes	406	10.7
	No	2966	77.9		No	3328	87.4
	No answer	73	1.9		No answer	73	1.9
Increased number of holidays	Yes	1157	30.4	Expanding welfare benefits	Yes	1067	28.0
	No	2577	67.7		No	2667	70.1
	No answer	73	1.9		No answer	73	1.9

Table S2. Cross tabulations of job satisfaction against age or career as dental hygienists.

		Age group						
		30<	30-39	40-49	50-59	60-69	70-	p-value
Do you wish to continue working as a dental hygienist?	Yes	1041	1248	2332	1961	389	13	<0.001
	No	84	67	109	123	64	8	
Do you feel that dental hygienist work is rewarding?	Strongly agree	216	264	529	459	109	9	<0.001
	Agree	751	886	1660	1457	315	11	
	Disagree	143	147	223	140	25	2	
	Strongly disagree	15	16	20	9	1	0	
		**Career as dental hygienists**						
		0-5	6-10	11-20	21-30	31-40	41-	p-value
Do you wish to continue working as a dental hygienist?	Yes	1026	729	1024	807	14	3007	<0.001
	No	51	30	69	66	9	209	
Do you feel that dental hygienist work is rewarding?	Strongly agree	215	147	249	159	11	717	<0.001
	Agree	750	531	712	577	11	2223	
	Disagree	96	69	119	122	2	238	
	Strongly disagree	8	9	13	13	0	15	

P-values were calculated by chi-square tests.

Table S3. Results of Item response theory analysis by two parameter logistic model

	Item discrimination (a_j)		Item difficulty (b_j)	
	Estimate	SE	Estimate	SE
Improvement in working conditions including higher salary	1.140	0.080	-0.105	0.037
Reduction of work volume	0.712	0.070	2.123	0.185
Improvement in interpersonal relations in the workplace	0.422	0.060	3.682	0.496
Reduction of working hours	0.882	0.081	1.763	0.134
Increased number of holidays	0.956	0.075	0.992	0.071
Expanding childcare support	0.823	0.078	2.676	0.214
Evaluation of expertise / qualifications	0.929	0.078	1.651	0.115
Opportunities for upgrading skills	0.501	0.062	2.393	0.279
Implementation of various working patterns and working hours	1.057	0.092	2.399	0.161
Strengthening workplace safety	0.808	0.074	1.977	0.154
Ensuring employment stability	0.842	0.082	2.807	0.230
Expanding welfare benefits	0.927	0.072	1.159	0.081

Table S4. Cross tabulations of motivation of Japanese dental hygienists and improvement in working conditions

		Do you wish to continue working as a dental hygienist?		P-value
		Yes	No	
Improvement in working conditions including higher salary	No	1680	94	0.171
	Yes	1828	124	
Reduction of work volume	No	2819	154	0.001
	Yes	689	64	
Improvement in interpersonal relations in the workplace	No	2887	159	0.001
	Yes	621	59	
Reduction of working hours	No	2819	140	<0.001
	Yes	689	78	
Increased number of holidays	No	2443	129	0.001
	Yes	1065	89	
Expanding childcare support	No	3081	186	0.275
	Yes	427	32	
Evaluation of expertise / qualifications	No	2755	184	0.039
	Yes	753	34	
Opportunities for upgrading skills	No	2634	187	<0.001
	Yes	874	31	
Implementation of various working patterns and working hours	No	3138	194	0.830
	Yes	370	24	
Strengthening workplace safety	No	2817	182	0.250
	Yes	691	36	
Ensuring employment stability	No	3128	194	0.935
	Yes	380	24	
Expanding welfare benefits	No	2514	148	0.231
	Yes	994	70	

P-values were calculated by chi-square tests.

Table S5. Results of factor analysis for the fourteen items that Japanese dental hygienists wish to improve

	Factor			
	1	2	3	4
Expanding welfare benefits	0.987	-0.010	-0.007	0
Reduction of working hours	0.062	0.541	-0.258	-0.100
Reduction of work volume	0.017	0.434	-0.155	-0.170
Increased number of holidays	0.147	0.393	-0.082	0.081
Implementation of various working patterns and working hours	0.087	0.335	0.018	0.119
Improvement in working conditions including higher salary	0.193	0.313	0.123	-0.049
Improvement in interpersonal relations in the workplace	0.033	0.157	0.082	0.030
Evaluation of expertise / qualifications	0.130	0.229	0.454	-0.102
Opportunities for upgrading skills	0.158	0.034	0.405	-0.042
Strengthening workplace safety	0.191	0.143	0.343	-0.090
Ensuring employment stability	0.174	0.131	0.185	0.056
Expanding childcare support	0.077	0.288	0.087	0.399
Total	0.96	0.881	0.803	0.428
Variance %	7.996	7.345	6.913	3.569
Cumulative variance %	7.996	15.341	22.254	25.823

Factor analysis by maximum likelihood method with Varimax rotation was carried out for 12 items that concern with the volition of dental hygienists.

Table S6. Cross tabulation of improvement in working conditions against financial reward

		Do you feel the work of dental hygienist is rewarding?				P-value
		Strongly agree	Agree	Disagree	Strongly disagree	
Improvement in working conditions including higher salary	No	415	1187	155	11	<0.001
	Yes	257	1380	277	31	
Reduction of work volume	No	560	2053	320	30	0.001
	Yes	112	514	112	12	
Improvement in interpersonal relations in the workplace	No	611	2116	283	27	<0.001
	Yes	61	451	149	15	
Reduction of working hours	No	556	2063	304	27	<0.001
	Yes	116	504	128	15	
Increased number of holidays	No	506	1779	260	19	<0.001
	Yes	166	788	172	23	
Expanding childcare support	No	603	2257	364	32	0.006
	Yes	69	310	68	10	
Evaluation of expertise / qualifications	No	578	2021	301	26	<0.001
	Yes	94	546	131	16	
Opportunities for upgrading skills	No	549	1916	319	27	<0.001
	Yes	123	651	113	15	
Implementation of various working patterns and working hours	No	617	2303	369	33	0.001
	Yes	55	264	63	9	
Strengthening workplace safety	No	575	2080	304	30	<0.001
	Yes	97	487	128	12	
Ensuring employment stability	No	622	2291	363	33	<0.001
	Yes	50	276	69	9	
Expanding welfare benefits	No	502	1843	282	24	0.001
	Yes	170	724	150	18	

P-values were calculated by chi-square tests.
